# Ovarian tachykinin signaling system induces the growth of secondary follicles during the gonadotropin-independent process

**DOI:** 10.1016/j.jbc.2025.108375

**Published:** 2025-03-03

**Authors:** Tsuyoshi Kawada, Masato Aoyama, Shin Matsubara, Tomohiro Osugi, Tsubasa Sakai, Shinji Kirimoto, Satsuki Nakaoka, Yuki Sugiura, Keiko Yasuda, Honoo Satake

**Affiliations:** 1 Bioorganic Research Institute, Suntory Foundation for Life Sciences, Kyoto, Japan; 2 Department of Chemistry, Biology, and Environmental Science, Faculty of Science, Nara Women's University, Nara, Japan; 3 Graduate School of Medicine and Faculty of Medicine, Kyoto University, Kyoto, Japan

**Keywords:** tachykinin, secondary follicle, growth, receptor, prostaglandin

## Abstract

Mammalian follicle growth development is mainly regulated by the hypothalamus–pituitary–gonadal axis after puberty. Although pituitary hormones, gonadotropins, are involved in hypothalamus–pituitary–gonadal axis signaling, they are not responsible for the growth of early stage follicles, namely, primordial follicles, primary follicles, and secondary follicles, in both sexually immature and mature individuals. Unlike those of gonadotropin-dependent follicle growth, the specific regulatory factors of gonadotropin-independent follicle growth have yet to be identified. Here, we identified tachykinins (TKs) as inducers of gonadotropin-independent secondary follicle growth. TKs play various roles as neuropeptides or hormones in a wide variety of biological events both in the central nervous system and in peripheral tissues, but a direct effect of TKs on ovarian follicles has yet to investigated. Follicle development was suppressed in sexually immature 3-week-old KO mice of *Tac1* gene encoding TKs (substance P and neurokinin A), which is independent of gonadotropins. TKs and their receptors are specifically localized to granulosa cells in mouse secondary follicles. Furthermore, TKs upregulate the prostaglandin (PG) synthase cyclooxygenase 2 *via* the Janus kinase 1–signal transducers and activators of transcription protein 3 signaling cascade. We also demonstrated that PGE2 and PGF2α are major PGs in the immature ovary, and the secondary follicle growth was enhanced by interaction between PGE2–PGF2α and their receptors, PGE2 receptor localized in the oocyte membrane and PGF2α receptor localized in the oocyte membrane, granulosa cells, and theca cells. Consequently, this study paves the way for exploring gonadotropin-independent early stage follicle growth systems and relevant dysfunctions, including pediatric endocrinological diseases.

Oogenesis is an essential process for species continuity. In mammals, there are six types of follicles that correspond with different developmental stages: primordial follicles, primary follicles, secondary follicles, preantral follicles, antral follicles, and Graafian follicles (1, 2, 3, 4, 5). Follicle growth and maturation are multistep biological events involving various endogenous and exogenous factors (1, 2). In particular, gonadotropins such as luteinizing hormone (LH) and follicle-stimulating hormone (FSH) are key factors for the growth and maturation of preantral follicles and antral follicles (1, 2, 3, 4, 5). Mammalian follicle growth development is regulated mainly by the hypothalamus–pituitary–gonadal (HPG) axis after puberty (6, 7). In adults, gonadotropin-releasing hormone (GnRH) is secreted from the hypothalamus to the pituitary gland and stimulates the release of LH and FSH from the pituitary into the blood (6, 7). Subsequently, gonadotropins are transported to the ovary *via* the circulatory system and act on follicles in the ovary (6, 7). LH stimulates theca cells in preantral follicles to produce testosterone, and estrogen is produced from testosterone in granulosa cells stimulated by FSH (6, 7). An increase in estrogen levels triggers the transition of preantral follicles to antral follicles. However, gonadotropins are not responsible for the growth of early stage follicles, such as primordial follicles, primary follicles, or secondary follicles. Notably, such premature gonadotropin-independent follicles grow at the fetal, neonatal, and infancy stages, at which point gonadotropins are not secreted; thus, the growth of gonadotropin-independent follicles is a critical step for generating high-quality fertile oocytes after puberty. Secondary follicles are composed of an oocyte, zona pellucida, granulosa cell layers, a basal lamina, and intracellular and extracellular theca cell layers. The major changes during the transition from secondary follicles to preantral follicles include oocyte enlargement, an increase in granulosa cell layers, and theca cell proliferation. Several oocytic factors, such as growth differentiation factor 9 and bone morphogenetic protein 15, and the granulosa cell and theca cell factor insulin-like growth factor 1, are thought to be involved in secondary follicle growth (2, 8, 9, 10, 11). However, these growth factors play a role in almost all stages of follicle development (2, 8, 9, 10, 11). Thus, no regulatory factor specific to the transition of secondary follicles to preantral follicles has been identified.

Tachykinins (TKs) are a large neuropeptide family widely conserved in chordates. In mammals, TKs play various roles as neuropeptides or hormones in a wide variety of biological events, including pain transmission, inflammation, cancer, depressive disorder, immune system, gut function, hematopoiesis, and reproduction (12, 13, 14). Mammalian TK family peptides are composed of four peptides: substance P (SP), neurokinin A (NKA), neurokinin B (NKB), and hemokinin-1/endokinin (12, 13, 14, 15, 16, 17). SP, NKA, and NKB exhibit favorable affinities for their receptors TACR1, TACR2, and TACR3, respectively (12, 13, 14, 15, 16, 17). Several reports have shown that TKs are involved in reproductive functions. NKB controls the secretion of kisspeptin from KNDy neurons in the hypothalamus, leading to the stimulation of GnRH neurons in mammals (7, 18). Loss of the *Tac1* gene, which encodes SP and NKA, delays the onset of puberty in mice (19). Furthermore, peripheral TKs were reported to stimulate uterine contractions (20) and enhance sperm motility (21). In contrast, the functions of ovarian TKs have not yet been determined except for their crucial role in inducing the growth of early stage ovarian follicles in the protovertebrate chordate *Ciona intestinalis* type A (22, 23, 24). Moreover, genes encoding TKs (SP, NKA, and NKB) and their receptors (TACR1–3) are expressed in the mammalian ovary (25, 26, 27), suggesting some biological role in reproductive functions in mammals.

In the present study, we revealed that mouse ovarian TKs promote secondary follicle growth in a gonadotropin-independent manner (namely, the HPG axis–independent stage).

## Results

### Follicle growth in the ovaries of Tac1 KO mice

**Figure 1:**
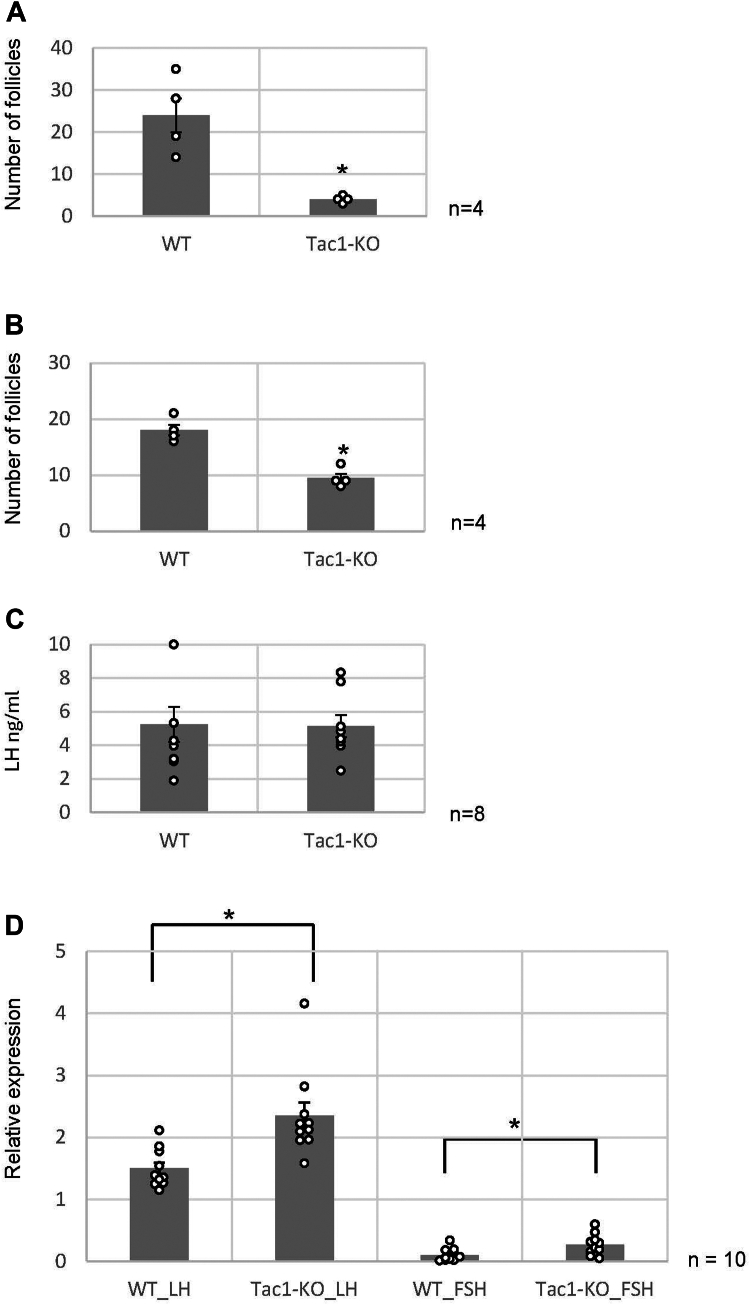
**Growth of follicles in the ovaries of *Tac1*-KO mice.***A*, estimation of the number of preantral or more-grown follicles (greater than 150 μm diameter) in a pair of ovaries of 3-week-old wildtype mice or *Tac1*-KO mice. The data are presented as the mean ± SEM. n = 4 per group. *B*, estimation of the number of antral follicles (with more than 300 μm in diameter) in a pair of ovaries from 8-week-old wildtype mice or *Tac1*-KO mice. The data are presented as the mean ± SEM. n = 4 per group. *C*, serum LH levels in 3-week-old wildtype and *Tac1*-KO mice. The data are presented as the mean ± SEM. n = 8 per group. *D*, real-time PCR-based quantification of *LH* and *FSH* gene expression in the ovaries of wildtype and *Tac1*-KO mice. *LH* and *FSH* gene expression was calculated from the ΔCt values using *GAPDH* as a housekeeping gene. Relative expression score was calculated as 2^−ΔΔCT^. The data are presented as the mean ± SEM. n = 10 per group. Significant differences (*p* < 0.05 according to the *t* test) are indicated by *asterisks*. FSH, follicle-stimulating hormone; LH, luteinizing hormone.

The ovaries were collected from 3-week-old and 8-week-old wildtype mice and *Tac1*-KO mice. Three-week-old mice are sexually immature, whereas 8-week-old mice are sexually mature and fertile. The follicle count revealed that the number of preantral or more-grown follicles (greater than 150 μm in diameter) in the ovaries of *Tac1*-KO mice was significantly decreased compared with that in the ovaries of wildtype mice; the percentage of these follicles in the KO group was approximately 16.7% (Fig. 1*A*, Table 1, and Movies S1 and S2). Similarly, we counted the antral follicles (greater than 300 μm in diameter) in the ovaries from 8-week-old mouse, revealing that the number of full-growth antral follicles in the ovaries of *Tac1*-KO mice was still approximately 50% less than that in the ovaries of wildtype mice (Fig. 1*B*, Table 1, and Movies S3 and S4). In addition, no difference in the size or number of litters was detected between the wildtype mice and *Tac1*-KO mice. These results suggest that TKs, rather than the HPG axis, promote the growth of premature follicles.

**Table 1:** Number of preantral and more grown follicles in the ovaries of 3-week-old and 8-week-old mice

	Number of follicles (>150 μm diameter; 3 wk)	Number of follicles (>300 μm diameter; 8 wk)
Wildtype	24.0 ± 4.0	18.0 ± 0.9
*Tac1*-KO	4.0 ± 0.4	9.5 ± 0.8

SP and NKA in the brain are thought to be involved in the stimulation of gonadotropins (FSH and LH) in adult mice (28, 29). Therefore, the suppression of follicle development in 3-week-old *Tac1*-KO mice (Fig. 1*A*, Table 1, and Movies S1 and S2) may be a result of reduced FSH and LH secretion because of the loss of TKs. To examine the effect of *Tac1* loss on the production and secretion of FSH and LH, we measured the gonadotropin levels in 3-week-old wildtype and *Tac1*-KO mice. The ELISA results demonstrated no difference in serum LH levels between 3-week-old wildtype and *Tac1*-KO mice (Fig. 1*C* and Table S1). Moreover, no differences in serum FSH levels were detected by ELISA in either 3-week-old wildtype or *Tac1*-KO mice (Table S1), and overall, the FSH levels were low, indicating that almost no FSH is secreted into the circulatory system of 3-week-old mice. Real-time PCR demonstrated that the LH and FSH gene expression in the pituitary glands of 3-week-old Tac1-KO mice was expressed 1.55-fold and 3.71-fold greater than in that of 3-week-old wildtype mice, respectively (Fig. 1*D*). Collectively, these ELISA and real-time PCR results confirmed that the suppression of follicle development in 3-week-old *Tac1*-KO mice (Fig. 1*A* and Table 1) was not because of a reduction in LH and FSH production or secretion. Moreover, these results suggest that TKs participate in the transition of secondary follicles to preantral follicles in sexually immature mice.

### Effects of TKs on follicle growth in the mouse ovary

**Figure 2:**
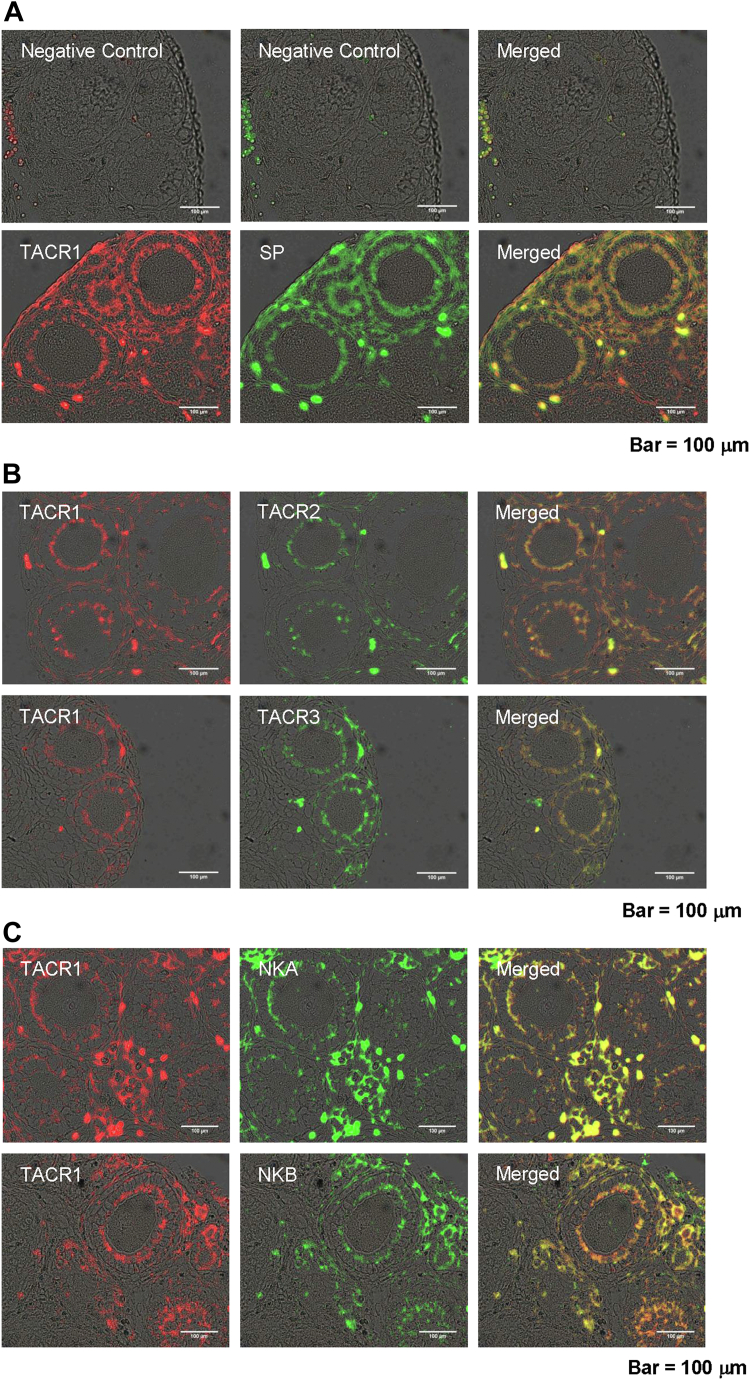
**Distribution of TKs and their receptors in the ovaries of 2-week-old mice.***A*, immunostaining of TACR1 (*red signal*) and SP (*green signal*) in an ovarian tissue section. The merged image shows the colocalization of TACR1 cells and SPs (*yellow signal*). No positive signal was observed for any of the antigens (*upper panel*). *B*, immunostaining of TACR1 (*red signal*) in an ovarian tissue section and another tachykinin receptor (TACR2 or TACR3; *green signal*). The merged image shows the localization of TACR1 and TACR2 cells or their colocalization (*upper panel*; *yellow signal*) or the colocalization of TACR1 and TACR3 cells (*lower panel*; *yellow signal*). *C*, immunostaining of TACR1 (*red signal*) and a TK (NKA or NKB; *green signal*) in an ovarian tissue section. The merged image shows the colocalization of TACR1 and NKA (*upper panel*; *yellow signal*) or the colocalization of TACR1 and NKB (*lower panel*; *yellow signal*). The scale bars indicate 100 μm. NKA, neurokinin; NKB, neurokinin B; SP, substance P; TK, tachykinin.

Initially, we examined the localization of SP, NKA, NKB, and their receptors (TACR1, TACR2, and TACR3) in the ovaries of 2-week-old mice that were sexually immature. Immunostaining of the ovarian tissue sections revealed that three TKs and their receptors were mainly localized in the inner granulosa cell layer of the secondary follicles (Fig. 2). Moreover, double immunostaining of ovarian tissue sections showed that the TACR1 signals largely overlapped with those of other TK receptors (TKRs, namely, TACR2 and TACR3) and TKs (Fig. 2), indicating that the three TKs and their receptors are all colocalized in the inner granulosa cell layer of the secondary follicles. Moreover, we detected SP by immunoelectron microscopy using the 2-week-old mouse ovary. SP was shown to be present in secretory vesicles near the Golgi apparatus of granulosa cells within secondary follicles (Fig. S1). Combined with the *Tac1* gene expression in the mouse ovary (25), these results suggested that SP is produced in the granulosa cells and secreted from the granulosa cells. Taken together, these immunohistochemical data indicated that SP, NKA, and NKB function in granulosa cells in an autocrine–paracrine manner at the secondary follicle stage.

**Figure 3:**
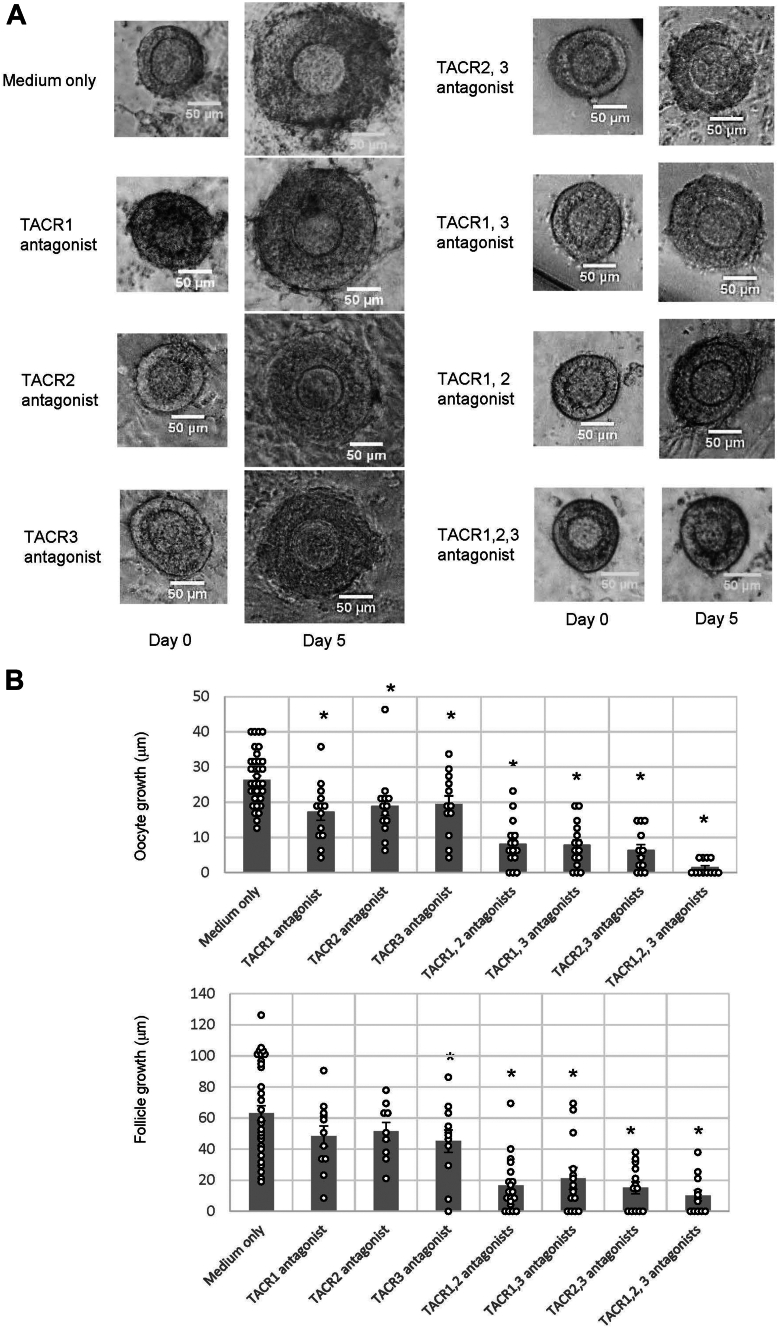
**Effects of TK antagonists on secondary follicle growth in three-dimensional follicle culture.***A*, secondary follicles were cultured in a collagen gel with theca cells for 5 days. The follicles were untreated (medium only) or treated with TKR antagonists (1 μM). The scale bars indicate 50 μm. *B*, the size of the follicles in three-dimensional culture in the presence or the absence of TKR antagonists for 5 days was measured. Oocyte growth was calculated by subtracting the oocyte diameter on day 0 from the oocyte diameter on day 5. Similarly, follicle growth was calculated by subtracting the follicle diameter length on day 0 from the follicle diameter length on day 5. Significant differences (*p* < 0.05 *versus* the medium-only group according to *t* test) are indicated by *asterisks*. TK, tachykinin; TKR, TK receptor.

To examine whether TKs are involved in the growth of secondary follicles in mice, secondary follicles were treated with the TACR1 antagonist L-703,606, the TACR2 antagonist GR-94800, and/or the TACR3 antagonist SB218795. Figure 3 shows the effect of the antagonists on secondary follicle growth. In the absence of the antagonists, the secondary follicles grew normally; the follicle diameter increased to 63.04 ± 4.83 μm after 5 days. Moreover, the oocyte diameter increased to 26.20 ± 1.27 μm after 5 days. Notably, treatment of the secondary follicles with all three antagonists mostly (approximately 85%) blocked follicle and oocyte growth. Subsequently, we examined the effect of each TK antagonist on follicle and oocyte growth. As shown in Figure 3, follicle and oocyte growth was moderately (20–30%) inhibited in the presence of each TKR antagonist. Cotreatment with two TKR antagonists inhibited the growth of follicles and oocytes by 65 to 75% (Fig. 3). Collectively, these biological assays revealed that TKs are responsible for the growth of follicles and oocytes at the secondary follicle stage and that SP, NKA, and NKB exhibit equipotent and additive effects on mouse secondary follicle and oocyte growth.

### Tachykininergic follicle growth *via* cyclooxygenase 2 gene expression in mice

**Figure 4:**
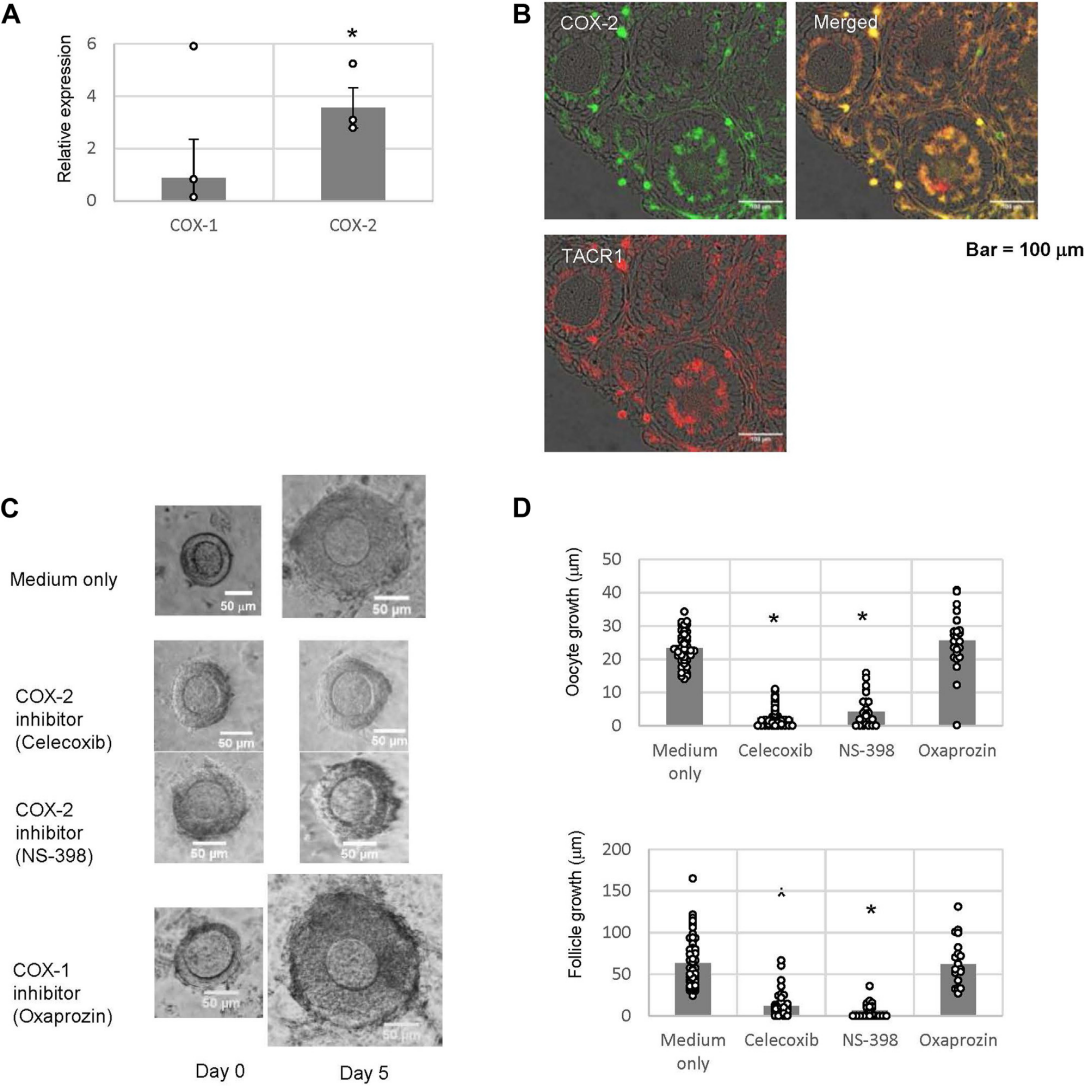
**PGs in the tachykininergic follicle growth system.***A*, real-time PCR-based quantification of *COX* gene expression in ovaries of mice treated with TACR1 agonist ([Sar9, Met(O2)11]-SP), TACR2 agonist (GR-64349), and TACR3 agonist (succinyl-[Asp, N-Me-phe8]sp.6-11) (each agonist at 1 μM) and in ovaries treated with the TACR1 antagonist (L-703,606), TACR2 antagonist (GR-94800), and TACR3 antagonist (SB218795) (each antagonist at 1 μM). The induction level of gene expression was calculated from the ΔΔCt values using the *β-actin* gene in the presence of TKR agonists or antagonists. Relative expression score was calculated as 2^−ΔΔCT^. Each point represents the mean ± SEM of three independent experiments. A significant difference between the TKR agonist–treated group and the antagonist-treated group (*p* < 0.05 according to the *t* test) is indicated by an *asterisk*. *B*, localization of COX-2 in the ovaries of 2-week-old mice. Immunostaining of COX-2 (*green signal*) and TACR1 (*red signal*) in an ovarian tissue section. The merged image shows the colocalization of COX-2 and TACR1 (*yellow signal*). The scale bars indicate 100 μm. *C*, effects of COX inhibitors on secondary follicle growth in three-dimensional follicle culture. Secondary follicles were cultured in a collagen gel with theca cells for 5 days. The follicles were untreated (medium only) or treated with 1 μM celecoxib (a COX-2 inhibitor), 5 μM NS-398 (a COX-2 inhibitor), or 2.2 μM oxaprozin (a COX-1 inhibitor). The bars indicate 50 μm. *D*, the size of the follicles grown in three-dimensional culture in the presence of each inhibitor for 5 days was measured. Oocyte growth was calculated by subtracting the oocyte diameter on day 0 from the oocyte diameter on day 5. Similarly, follicle growth was calculated by subtracting the follicle diameter length on day 0 from the follicle diameter length on day 5. Significant differences (*p* < 0.05 *versus* medium-only group according to the *t* test) are indicated by *asterisks*. COX, cyclooxygenase; PG, prostaglandin.

To identify TK-induced factors involved in the growth of secondary follicles, we screened for differentially expressed genes in the ovaries of 2-week-old mouse treated with all the TKR antagonists and 2-week-old mouse treated with TKR agonists. Gene expression profile analysis demonstrated that the expression of *COX-2* (cyclooxygenase 2), a gene encoding a prostaglandin (PG) H synthase (30, 31), was downregulated by treatment with the three antagonists (accession no.: GSE213246, Table S2). Real-time PCR also confirmed 3.6-fold increase in *COX-2* gene expression in the ovaries of mice treated with TK agonists (Fig. 4*A*). Likewise, real-time PCR confirmed significant decrease of *COX-2* gene expression in the ovaries of 2-week-old *Tac1-KO* mice, compared with those in the wildtype (Fig. S2). These results revealed that TKs induced *COX-2* gene expression in 2-week-old mouse ovaries. In addition, the expression of *COX-1*, a subtype of *COX-2* that is constitutively expressed (31, 32), was not altered by TKR agonist–antagonist treatment. Subsequently, we investigated the expression and localization of COX-2 in the ovaries of 2-week-old mice. Immunostaining demonstrated the specific colocalization of TACR1 and COX-2 in the inner granulosa cell inner layer of secondary follicles (Fig. 4*B*). Moreover, the colocalization of TACR1 with TACR2 and TACR3 (Fig. 2) indicated that COX-2 colocalizes with all three TKRs. Combined with the induction of *COX-2* gene expression by TKR agonists (Fig. 4*A*), these results verified that TK induces *COX-2* gene expression in the granulosa cells of secondary follicles. Furthermore, follicle growth was inhibited by 82% and 90% when follicles in three-dimensional culture were treated with the COX-2 inhibitors, NS-398 and celecoxib, respectively. Moreover, oocyte growth was inhibited by 82% and 90% after treatment with NS-398 and celecoxib (Fig. 4, *C* and *D*). In contrast, the secondary follicles grew normally in the presence of the COX-1-specific inhibitor oxaprozin (Fig. 4, *C* and *D*), revealing that COX-1 is not involved in follicle or oocyte growth. Taken together, these results indicated that COX-2 plays a crucial role in TK-induced secondary follicle formation.

COX-2 synthesizes PG H2, which is a basal precursor for various PGs, PGD, PGE2, PGF2α, PGI, and thromboxane A (30, 31) (Fig. S3), suggesting that some of these PGs are involved in TK-induced secondary follicle development. Of note, liquid chromatography‒mass spectrometry demonstrated that PGE2 and PGF2α are present in the ovaries (Fig. S4). Moreover, only the *PGE receptor 2* (*EP2*) gene, the *PGE receptor 4* (*EP4*) gene, and the *PGF receptor* (*FP*) gene were found to be expressed in the ovaries of 2-week-old mice (Fig. S3), and the *PGE2 synthase* gene and *PGF2α synthase* gene were also expressed in the ovaries of 2-week-old mice (Fig. S3). These results indicated that PGE2 and PGF2α were major PGs upregulated by TKs in 2-week-old mouse ovaries, and that these PGs play important roles in follicle development *via* the PGE2-EP2, PGE2-EP4, and/or PGF2α-FP signaling cascades.

**Figure 5:**
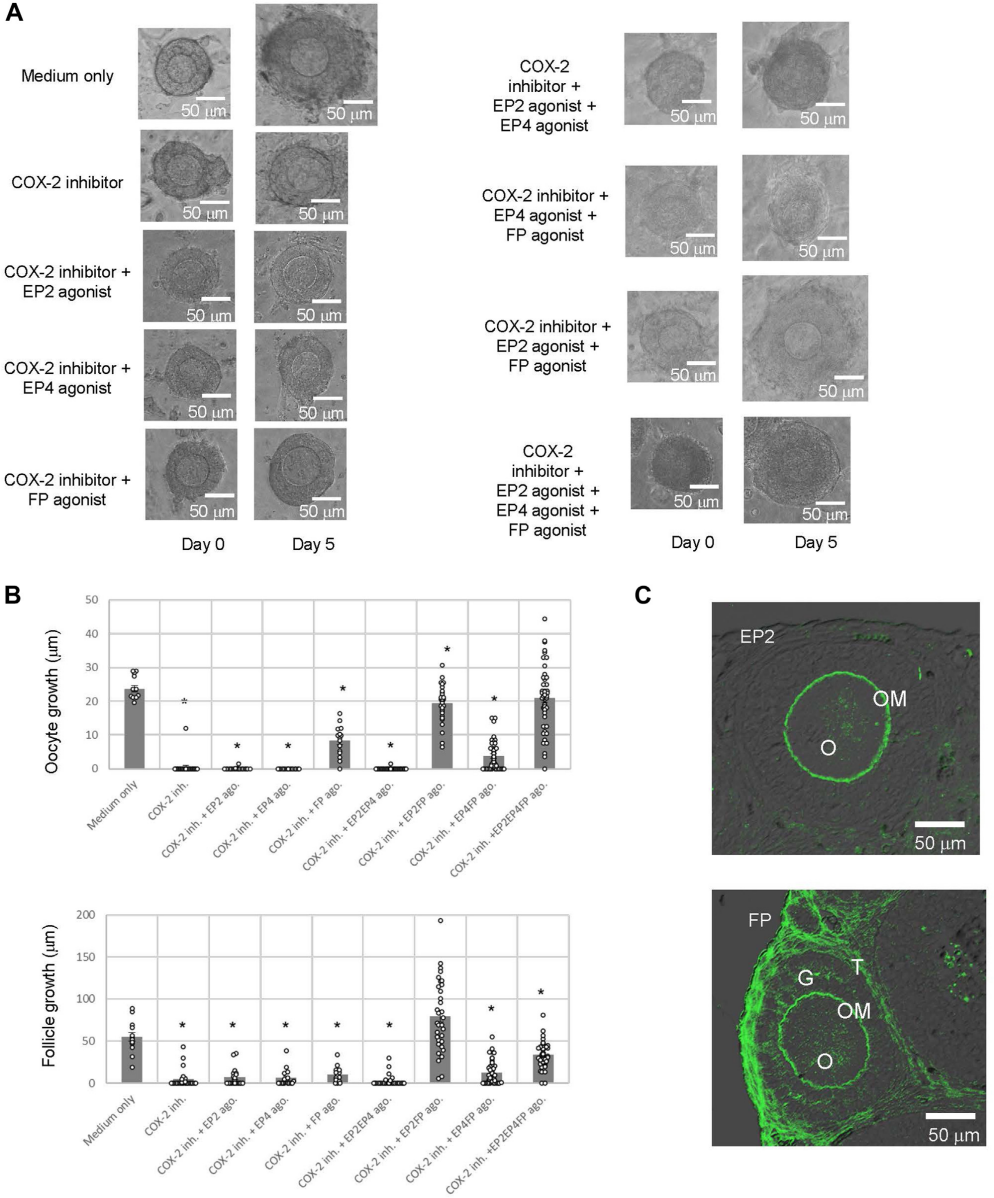
**PGE2 and PGF2α in the tachykininergic follicle growth system.***A*, secondary follicles were cultured in a collagen gel with theca cells for 5 days. In addition to 1 μM celecoxib (a COX-2 inhibitor), the follicles were treated with one or more prostaglandin receptor agonists (the EP2 agonist Butaprost at 1 μM; the EP4 agonist CAY10598 at 10 μM; and the PGF2α agonist 17-phenyl trinor at 1 μM). The scale bars indicate 50 μm. *B*, the size of the follicles grown in three-dimensional culture in the presence of each inhibitor or agonist for 5 days was measured. Oocyte growth was calculated by subtracting the oocyte diameter on day 0 from the oocyte diameter on day 5. Similarly, follicular growth was calculated by subtracting the follicle diameter length on day 0 from the follicle diameter length on day 5. Significant differences (*p* < 0.05 *versus* medium-only group according to *t* test) are indicated by *asterisks*. *C*, localization of EP2 and FP in the ovaries of 2-week-old mice. Immunostaining of EP2 (*upper image*) and FP (*lower image*) in a section of the ovary. The scale bars indicate 50 μm. COX, cyclooxygebnase; EP2, PGE receptor 2; EP4, PGE receptor 4; FP, PGF receptor; G, granulosa cell; O, oocyte; OM, oocyte membrane; T, theca cell.

To clarify whether PG signaling cascades are involved in the promotion of secondary follicle growth by COX-2, we evaluated the recovery of secondary follicles impaired by celecoxib (a COX-2-specific inhibitor) *via* the use of an EP2-specific agonist (Butaprost), an EP4-specific agonist (CAY10598), and an FP-specific agonist (17-phenyl trinor PGF2α) using the same follicle growth assays (Fig. 5, *A* and *B*). Notably, treatment with Butaprost and the 17-phenyl trinor PGF2α resulted in almost normal secondary follicle development (144.8% follicle growth and 82.0% oocyte growth compared with follicle culture medium only) despite the presence of celecoxib (Fig. 5, *A* and *B*). These results demonstrated that the two agonists recovered growth-suppressed follicles (Fig. 5, *A* and *B*). On the other hand, the suppressive effect of celecoxib was not fully reversed by treatment with each single PG receptor agonist or other agonist pairs. Taken together, these results provide evidence that both EP2 and FP play major roles in the development of secondary follicles. Immunostaining revealed that EP2 was localized to the oocyte membrane of secondary follicles, whereas FP was present not only in the oocyte membrane but also in the granulosa cells and theca cells of secondary follicles (Fig. 5*C*). Taken together, these results revealed the essential pathway for TKergic secondary follicle development.

### Upregulation of the COX-2 gene by TKs during the growth of secondary follicles

**Figure 6:**
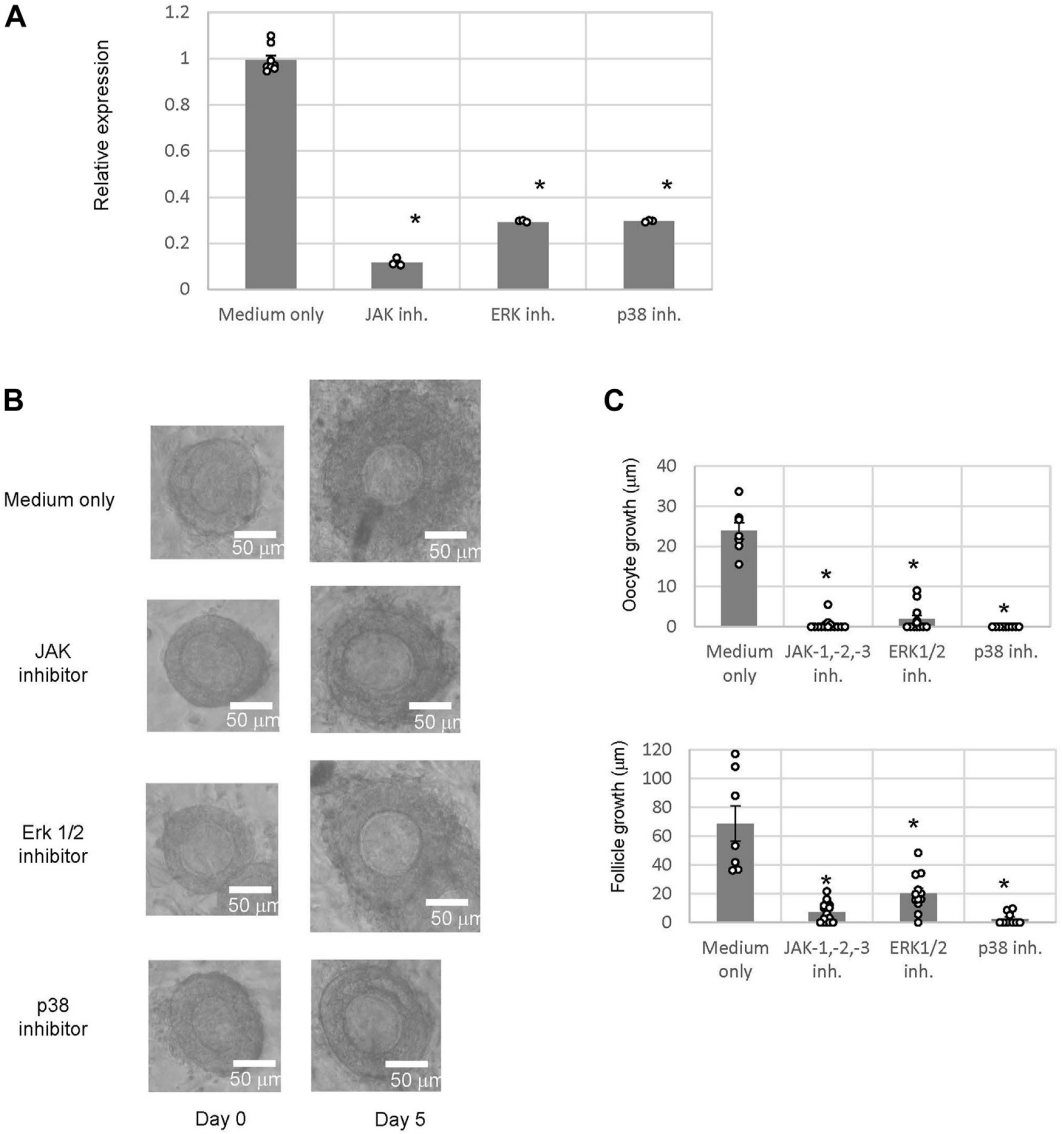
**The JAK–STAT signaling pathway in the tachykininergic follicle growth system.***A*, real-time PCR-based quantification of *COX-2* gene expression in mouse secondary follicles treated with phosphorylation inhibitors (JAK inhibitor, JAK inhibitor I at 40 μM; ERK1/2 inhibitor PD98059 at 100 μM; and p38 inhibitor SB202190 at 100 μM). The induction level of *COX-2* gene expression was calculated from the ΔCt values using the *β-actin* gene. Moreover, the degree to which *COX-2* gene expression was induced was calculated from the ΔΔCt values of the follicles treated with the COX-2 inhibitor and each phosphorylation inhibitor. Relative expression score was calculated as 2^−ΔΔCT^. Each point represents the mean ± SEM of three independent experiments. Significant differences (*p* < 0.05 *versus* medium-only group according to *t* test) are indicated by *asterisks*. *B*, effect of each phosphorylation inhibitor on secondary follicle growth in three-dimensional follicle culture. Secondary follicles were cultured in a collagen gel with theca cells for 5 days. The follicles were untreated (medium only) or treated with each phosphorylation inhibitor (JAK inhibitor, 40 μM; ERK1/2 inhibitor, 100 μM PD98059; or p38 inhibitor, 100 μM SB202190). The bars indicate 50 μm. *C*, the size of the follicles grown in three-dimensional culture in the presence of each phosphorylation inhibitor for 5 days was measured. Oocyte growth was calculated by subtracting the oocyte diameter on day 0 from the oocyte diameter on day 5. Similarly, follicle growth was calculated by subtracting the follicle diameter length on day 0 from the follicle diameter length on day 5. Significant differences (*p* < 0.05 *versus* medium-only group according to *t* test) are indicated by *asterisks*. COX, cyclooxygenase; ERK1/2, extracellular signal–regulated kinase 1/2; JAK, Janus kinase; STAT, signal transducers and activators of transcription protein.

Subsequently, we attempted to identify signaling cascades involved in *COX-2* gene upregulation by TKs. To date, TKs have been shown to upregulate *COX-2* gene expression *via* the phosphorylation of Janus kinases (JAKs) (33), extracellular signal–regulated kinase (ERK) 1/2, and p38 in nonovarian tissues or cells in humans and mice (34). To elucidate the signaling cascade involved in *COX-2* gene upregulation in secondary follicles, we evaluated *COX-2* gene expression levels by real-time PCR in secondary follicles treated with each phosphorylation inhibitor: a JAK inhibitor (JAK inhibitor I), an ERK1/2 inhibitor (PD98059), and a p38 inhibitor (SB202190). *COX-2* gene expression was downregulated by each phosphorylation inhibitor (Fig. 6*A*). Notably, the JAK inhibitor most potently suppressed *COX-2* gene expression in secondary follicles (Fig. 6*A*). Moreover, 5 days of treatment with these inhibitors resulted in the suppression of oocyte and follicle growth (Fig. 6, *B* and *C*). These results indicated that the phosphorylation of JAK, ERK1/2, and p38 participates in the induction of secondary follicle development *via* the upregulation of *COX-2* gene expression.

**Figure 7:**
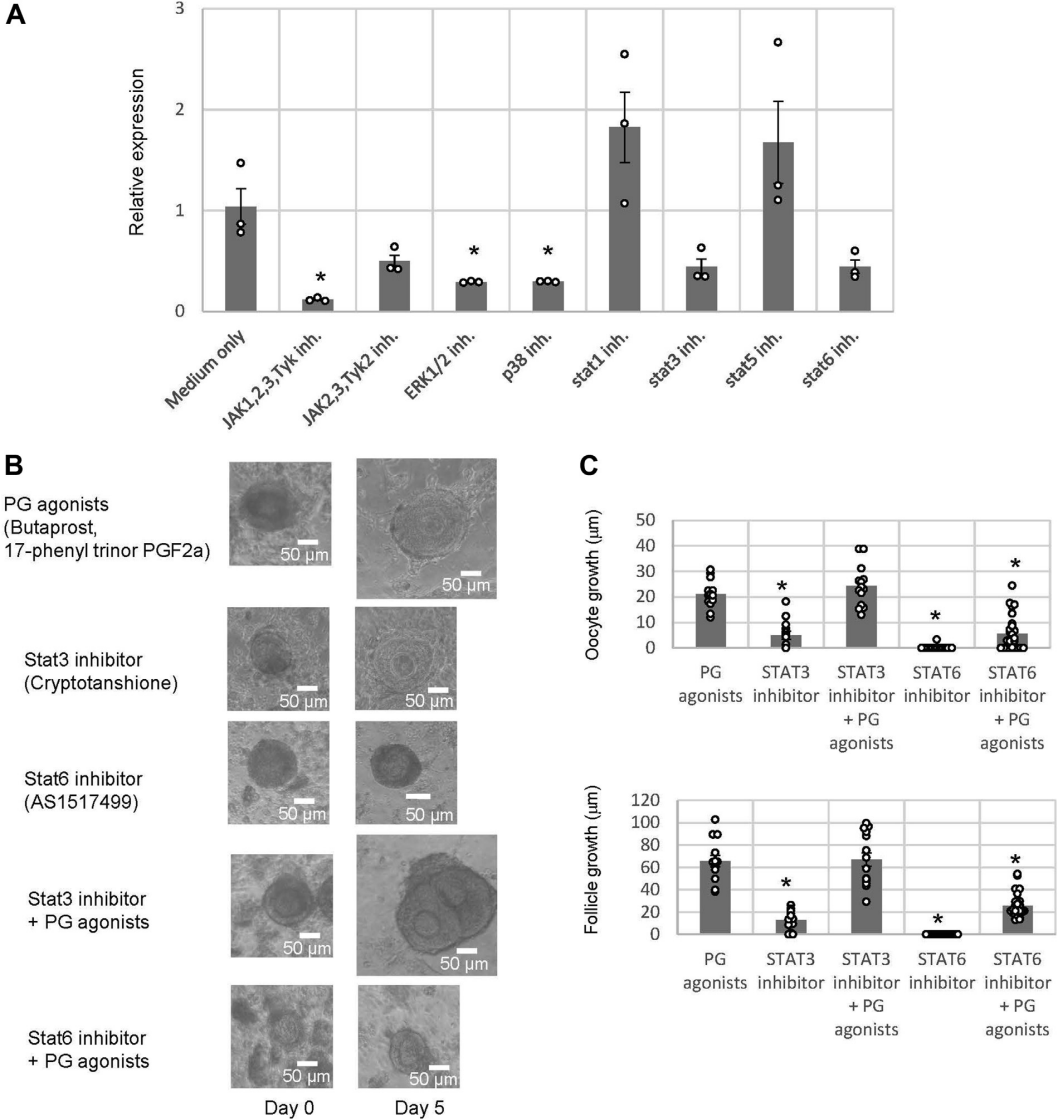
**STAT3 activation in the tachykininergic follicle growth system.***A*, real-time PCR-based quantification of *COX-2* gene expression in mouse secondary follicles treated with each JAK or STAT inhibitor (JAK1, 2, 3 and Tyk2 inhibitor, 40 μM JAK inhibitor I; JAK2, 3, and Tyk2 inhibitor, 50 nM pacritinib/1 nM tofacitinib citrate; STAT1 phosphorylation inhibitor, FAMP, 5.4 nM; STAT3 phosphorylation inhibitor, cryptotanshinone, 5 μM; STAT5 phosphorylation inhibitor, IQDMA, 0.8 μM; and STAT6 phosphorylation inhibitor, AS1517499, 10 μM). The induction level of *COX-2* gene expression was calculated from the ΔΔCt values using the *β-actin* gene in the presence of each inhibitor. As a positive control group, ΔΔCt values were subtracted between the follicles treated with no ligand and those treated with tachykinin antagonists. Relative expression score was calculated as 2^−ΔΔCT^. Each point represents the mean ± SEM of three independent experiments. Significant differences (*p* < 0.05 *versus* the PG agonist–treated group according to the *t* test) are indicated by *asterisks*. *B*, secondary follicles isolated from mouse ovaries were cultured in a collagen gel with theca cells for 5 days. The follicles were treated with PG agonists only, an STAT3 inhibitor only, an STAT6 inhibitor only, an STAT3 inhibitor and PG agonists, or an STAT6 inhibitor and PG agonists. The scale bars indicate 50 μm. *C*, the size of the follicles grown in three-dimensional culture in the presence or the absence of PG agonists and STAT inhibitors for 5 days was measured. Oocyte growth was calculated by subtracting the oocyte diameter on day 0 from the oocyte diameter on day 5. Similarly, follicle growth was calculated by subtracting the follicle diameter length on day 0 from the follicle diameter length on day 5. Significant differences (*p* < 0.05 *versus* the PG agonist–treated group according to the *t* test) are indicated by *asterisks*. COX, cyclooxygenase. JAK, Janus kinase; PG, prostaglandin; STAT, signal transducers and activators of transcription protein.

**Figure 8:**
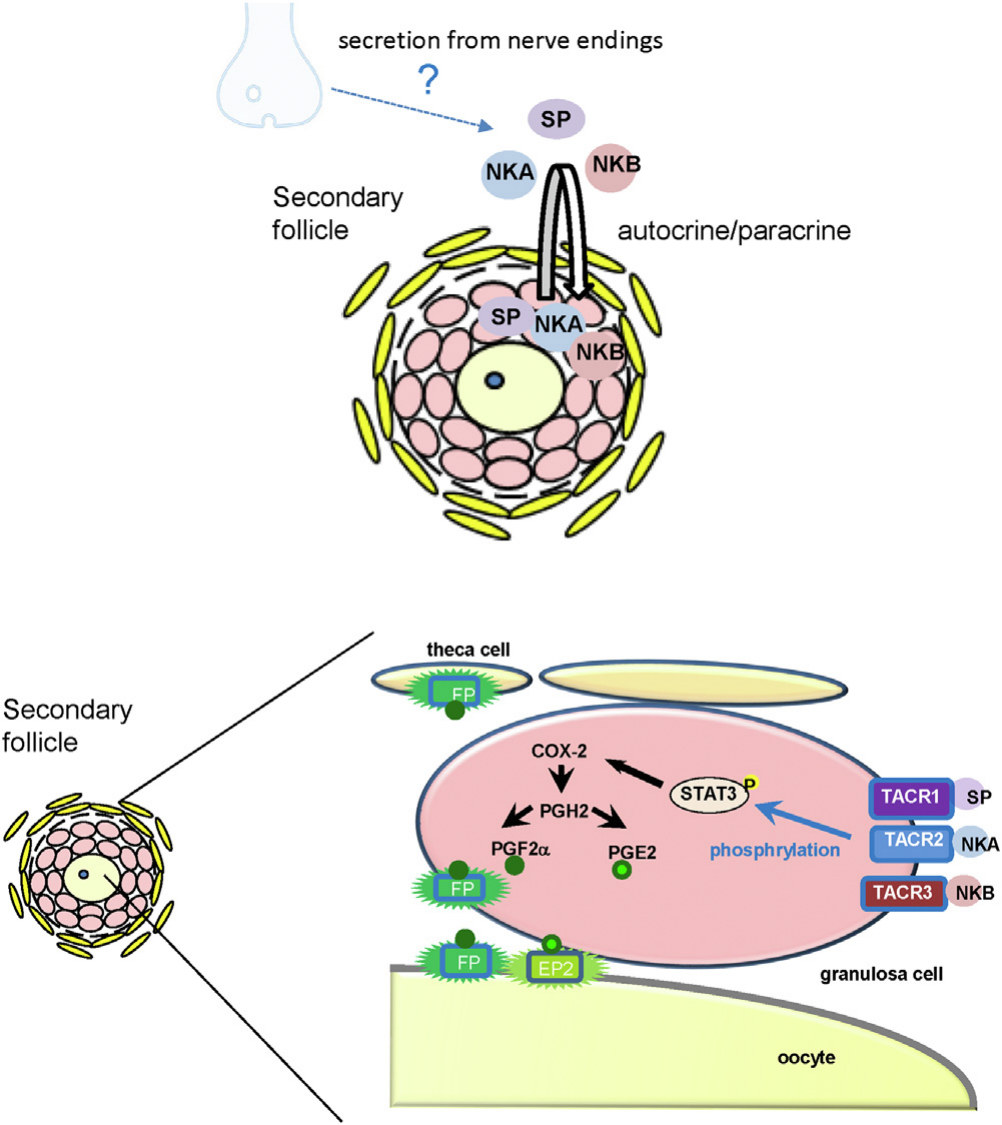
**Schematic image of TK-induced secondary follicle growth.** TKs (SP, NKA, and NKB) act on granulosa cells of the secondary follicle *via* autocrine–paracrine from granulose cells. In addition, SP, NKA, and NKB secreted from nerve endings may also participate in the secondary follicle growth. In granulosa cells, the interaction between TKs and their receptors induces *COX-2* gene expression *via* STAT3 phosphorylation, leading to the synthesis of PGE2 and PGF2α. PGE2 interacts with its receptor in oocytes, whereas PGF2α interacts with its receptor in oocytes, granulosa cells, and theca cells. Activation of the receptors in various cells induces secondary follicle growth in an integrated manner. The illustration was partially created using BioRender.com (https://www.biorender.com/). COX, cyclooxygenase; NKA, neurokinin A; NKB, neurokinin B; STAT3, signal transducers and activators of transcription protein 3; SP, substance P; TK, tachykinin.

JAKs activate the phosphorylation of signal transducers and activators of transcription proteins (STATs), which are essential for their translocation to the nucleus and transcriptional activity. Since four JAKs (JAK1, JAK2, JAK3, and Tyk2) and seven STATs (STAT1, STAT2, STAT3, STAT4, STAT5a, STAT5b, and STAT6) are present in mice, we initially investigated the gene expression of each *JAK* gene and *STAT* gene in secondary follicles to determine the JAK–STAT activation cascades required for secondary follicle growth. RT–PCR revealed that the *JAK1*, *STAT2*, *STAT3*, *STAT5a*, *STAT5b*, and *STAT6* genes were expressed in secondary follicles (Fig. S5). Subsequently, to elucidate the JAK–STAT cascades that induce *COX-2* gene expression, we evaluated *COX-2* gene expression in the presence of phosphorylation inhibitors of JAKs and STATs in secondary follicles. Results showed that *COX-2* gene real-time PCR showed that, compared with treatment with pacritinib–tofacitinib citrate (inhibitors of JAK2,3 and Tyk2 phosphorylation), treatment with JAK inhibitor I (an inhibitor of JAK1, 2, 3, and Tyk2 phosphorylation) strongly downregulated *COX-2* gene expression (Fig. 7*A*), indicating that phosphorylation of JAK1 plays a major role in *COX-2* gene expression in secondary follicles. Moreover, the real-time PCR expression was suppressed not by a STAT1 phosphorylation inhibitor (FAMP) or a STAT5 phosphorylation inhibitor (IQDMA) but by a STAT3 phosphorylation inhibitor (cryptotanshione) and a STAT6 phosphorylation inhibitor (AS1517499) (Fig. 7*A*). These results suggested that the phosphorylation of STAT3 and STAT6 is responsible for *COX-2* gene expression. Furthermore, we observed the recovery effect of Butaprost (an EP2 agonist) and 17-phenyl trinor PGF2α (an FP agonist) on secondary follicle suppression by an STAT3 inhibitor or an STAT6 inhibitor. The suppression of secondary follicle growth by the STAT3 inhibitor was completely reversed by the presence of the EP2 agonist and FP agonist (Fig. 7, *B* and *C*). These results proved that STAT3 phosphorylation *via* the activation of EP2 and FP is a prerequisite for secondary follicle growth. On the other hand, the impairment of secondary follicle growth by the STAT6 inhibitor was only approximately 35% reversed by treatment with the EP2 agonist or FP agonist (Fig. 7, *B* and *C*). Consequently, the phosphorylation of STAT6 is likely to be required to activate not only *COX-2* gene upregulation but also other signaling pathways for secondary follicle development. Taken together, these results verify the essential molecular mechanisms underlying TKergic secondary follicle development (Fig. 8), which include the following: 1) SP, NKA, and NKB activate the phosphorylation of JAK1–STAT3, leading to the upregulation of *COX-2* gene expression in granulosa cells and 2) the upregulation of *COX-2* gene expression increases the levels of PGE2 and PGF2α, which induce the growth of oocytes and follicles *via* interaction with EP2 on oocytes and with FP on oocytes, granulosa cells, and theca cells, respectively.

## Discussion

Ovarian follicles grow from the primordial to preantral stage prior to sexual maturation and gonadotropin release from the pituitary gland (1, 2). After puberty, gonadotropins induce follicle maturation from preantral follicles to antral follicles followed by ovulation, whereas follicle growth from primordial follicles to preantral follicles is not mediated by gonadotropin stimulation (1, 2). Consequently, the gonadotropin-independent follicle growth process at an early stage plays crucial roles in the entire reproductive system by regulating immature follicle quality during the entire lifespan of females. However, neither regulatory factors nor the underlying molecular mechanisms for gonadotropin-independent follicle growth have been identified, in contrast with those of gonadotropin-dependent follicle growth system. In the present study, we identified TKs as key factors for the induction of mouse secondary follicle growth, which is a gonadotropin-independent process. TKs are secreted from granulosa cells in mouse secondary follicles in an autocrine or a paracrine fashion, leading to the induction of secondary follicle growth *via* the TK—JAK1—STAT3—COX-2—PGE2/PGF2α signaling pathway (Fig. 8). Although most studies on follicle development have focused on sexually mature individuals, namely the HPG axis, the present study explored the endocrinology of sexually immature individuals, namely non–HPG axis process.

Several factors, such as transforming growth factor β family proteins, are thought to be involved in gonadotropin-independent follicle growth (8, 9, 35, 36). However, these factors were reported based on the use of granulosa cell or theca cell culture and the localization of these factors in the ovary (8, 9, 35, 36). Thus, no experimental evidence for the involvement of these factors in follicle growth has ever been provided. In the present study, we verified the biological role of TKs in the specific induction of secondary follicle growth (Fig. 3) using a three-dimensional follicle culture system (37). In other words, TKs were originally shown to be positive regulators of secondary follicle growth, which is a gonadotropin-independent reproductive stage.

TKs play roles in multiple biological processes, including reproduction (12, 13). For example, NKB is responsible for preantral and antral follicle growth and maturation *via* regulation of GnRH secretion from the hypothalamus (6, 7). SP and NKA are suggested to control the timing of puberty onset, and NKA stimulates LH release in the presence of sex steroids in mice (19, 28). Notably, these TKergic reproductive functions occur *via* the HPG axis, which is a canonical gonadotropin-dependent endocrine system. In contrast, gonadotropin gene expression and secretion were not affected in *Tac1* KO mice, regardless of follicle growth (Fig. 1). Moreover, TKs and TKRs were shown to be localized in the granulosa cells of secondary follicles (Fig. 2, *A* and *C*). Consistent with these results, RT–PCR results showed that genes encoding TKs and TKRs were all expressed in the mammalian ovary (25, 26, 27). Altogether, the present study indicates that secondary follicle growth is induced by TKs secreted from the granulosa cells of secondary follicles as peripheral paracrine–autocrine factors (Fig. 8). On the other hand, TKs were found to be in the nerve ending of the ovary (38). Therefore, the possibility cannot be excluded that TKs derived from the nerves also participate in the secondary follicle growth (Fig. 8). The specific and common biological roles of granulosa cell–derived TKs and nerve-derived TKs remain unclear. Future studies are required to elucidate the mechanisms underlying their respective contributions to follicular development.

Three TKs (SP, NKA, and NKB) and three TKRs (TACR1, 2, and 3) were expressed in the same granulosa cells within the mouse secondary follicles (Fig. 2), and activation of each TKR exhibited almost equivalent effects on secondary follicle growth (Fig. 3). Inhibition of all three TKRs completely blocked oocyte growth, whereas inhibition of each TKR reduced follicle growth by approximately two-thirds (Fig. 3). Similarly, the follicle growth was stepwisely suppressed by addition of each TKR antagonist (Fig. 3). These results demonstrated that the three TK/TKR signaling pathways have additive effects on secondary follicle growth (Fig. 3). In addition, these *in vitro* assays of secondary follicle growth are in accordance with the partial arrest of follicle growth in *Tac1*-KO mice (Fig. 1), in which NKB is still functional and stimulates all TKRs (typically TACR3 and less potently TACR1 and TACR2). Such equipotent biological effects of multiple homologous peptides and receptors are highly likely to at least partially ameliorate follicle development dysfunction that originates from gene mutations or impaired relevant signal transduction pathways. Thus, the present study revealed that TKergic secondary follicle growth can be compensated *via* reciprocal rescue of biological functions upon unexpected functional disruption of a TK peptide or receptor.

In the present study, PGE2 and PGF2α were shown to act as critical factors of TKergic secondary follicle growth (Figs. 4 and 5). TKs upregulate *COX-2* gene expression (Fig. 4*A*), leading to the production of PGE2 and PGF2α in the granulosa cells of secondary follicles (Fig. 4, *B* and *E*). Moreover, the coadministration of an EP2 (PGE2-specific receptor) agonist and an FP (PGF2α-specific receptor) agonist abrogated the complete suppression of follicle growth by the COX-2 inhibitor (Fig. 5, *A* and *B*). In contrast, the suppression of follicle growth by the COX-2 inhibitor was not abrogated by the administration of either the EP2 agonist or the FP agonist (Fig. 5, *A* and *B*). These results indicate that the activation of both EP2 and FP is indispensable for secondary follicle growth, unlike the additive effects of TKs (Fig. 3, *A* and *B*), and are in agreement with the findings of different signaling cascades involving EP2 and FP. EP2 is coupled to Gs, inducing the cAMP signaling pathway, whereas FP is coupled to Gq/11, triggering intercellular Ca^2+^ mobilization (39, 40). Moreover, the distribution of EP2 in the secondary follicle is different from that of FP. FP was localized to the oocyte membrane, granulosa cells, and theca cells, whereas EP2 was detected only in the oocyte membrane (Fig. 5*C*). Collectively, these results suggest that PGE2 and PGF2α exert multiple cooperative functions in oocytes, granulosa cells, and theca cells during TKergic secondary follicle growth. In contrast, the EP4 agonist mildly reduced the effect of the EP2 agonist and the FP agonist on the rescue of the growth of secondary follicles treated with the COX-2 inhibitor (Fig. 5, *A* and *B*). Consequently, the present study provides evidence that the intrafollicular PGE2–EP2 and PGF2α–FP signaling cascades play crucial roles in secondary follicle growth and suggests that PGE2–EP4 signaling is required for the suppression of secondary follicle overgrowth. Additional investigations of the molecular mechanisms underlying TK–PG-directed secondary follicular growth are underway.

In the antral follicle, PGE2 serves as a regulator of reproductive functions under the control of the HPG axis. Gonadotropins upregulate PGE2 production in cumulus cells (a type of granulosa cell in antral follicles), and the activation of EP2 by PGE2 induces cumulus cell structural disorganization, leading to cumulus expansion and ovulation (39, 41). These studies indicate that PGE2 acts merely on granulosa cell–related cells in the antral follicle. In contrast, EP2 was localized not to granulosa cells but to the oocyte membrane of secondary follicles (Fig. 5*C*). Combined with these findings, the present study substantiates that the biological role of PGE2 in secondary follicle growth (gonadotropin-independent stage) is distinct from that in antral follicles (gonadotropin-dependent stage). In other words, PGE2 plays pivotal roles not only in the maturation and ovulation of gonadotropin-dependent follicles but also in the growth of gonadotropin-independent follicles. PGF2α induces the lysis of the corpus luteum appearing after ovulation (42), whereas the role of PGF2α in gonadotropin-dependent follicles has not been fully elucidated. In contrast, PGF2α induces secondary follicle growth, suggesting that PGF2α mainly exerts its effects on follicles at the gonadotropin-independent stage rather than follicles at the gonadotropin-dependent stage.

*COX-2* gene expression is upregulated by TKs *via* the JAK1–STAT3 phosphorylation cascade in secondary follicles, leading to secondary follicle growth (Figs. 6 and 7). In human colonic epithelial cells, *COX-2* gene expression is also upregulated by SP *via* JAK–STAT phosphorylation (34). Intriguingly, *COX-2* gene expression in human colonic epithelial cells is upregulated by JAK2–STAT3 phosphorylation or JAK2–STAT5 phosphorylation but not by JAK1–STAT3 phosphorylation (34), indicating that the regulatory cascade for *COX-2* gene expression varies between granulosa cells of secondary follicles and colonic epithelial cells. In addition, *COX-2* gene expression is downregulated by STAT3 phosphorylation in hepatocellular carcinoma cells (43), demonstrating that *COX-2* gene expression is not necessarily upregulated by JAK–STAT signaling activation. These findings lead to the presumption that TKergic *COX-2* gene expression *via* JAK1–STAT3 phosphorylation is specific to granulosa cells in the secondary follicle.

Unlike vertebrates including mammals, invertebrates have acquired neither the HPG axis nor the gonadotropins and developed species-specific follicular development and ovulation systems. For example, oocyte maturation and ovulation are directly induced by neuropeptides, vasopressin, and cholecystokinin but not by gonadotropins in the ascidian *C. intestinalis* type A, which belongs to the phylum urochordate and is the most closely related sister group of vertebrates (44, 45, 46, 47). In previous studies, we also substantiated that *Ciona* vitellogenic follicle growth is induced by the *Ciona* TK homolog (CiTK) (22, 23, 24). Combined with the fact that *Ciona* is not endowed with gonadotropinergic follicle development systems, our present data suggest that TKergic follicle growth systems might have emerged at least in a common ancestor of vertebrates and urochordates before ancestral vertebrates acquired the HPG axis and gonadotropins. In contrast, CiTK was found to upregulate the expression of *cathepsin D* (24), which is an aspartic protease responsible for the processing of vitellogenin in fish and birds (48, 49). These findings suggest that CiTK-induced cathepsin D enhances the processing of vitellogenin, leading to vitellogenic follicle growth. In contrast, no vitellogenin has been found in mammals, suggesting that the contribution of cathepsin D to mammalian follicle growth is much lower than that of nonmammalian chordates and that the TKergic follicular development process might have transitioned from cathepsin D system to the activation of the PG cascade during mammalian evolution. However, the molecular mechanisms underlying tachykinergic follicle development in nonmammalian vertebrates awaits further study.

A further marked difference between the mouse and ascidian TKergic follicle growth systems lies in the tissues producing TKs. CiTK is produced in the central nervous system and is transported to the ovary in *Ciona* (22, 23, 24), indicating the presence of a TKergic neuroendocrine system. In contrast, mouse TKs are produced in the granulosa cells of secondary follicles (Fig. 2), demonstrating the autocrine secretion or paracrine secretion of TKs from granulosa cells. Taken together, these results indicate that *Ciona* TKergic vitellogenic follicle growth is regulated by the central nervous system, whereas mouse TKergic secondary follicle growth is directly induced by ovarian TKs in peripheral tissue. In other words, direct TKergic follicle growth might have evolved from “centralized” regulation to “local autonomy” regulation during the chordate evolutionary process. Overall, direct TKergic follicle growth systems likely emerged in common ancestors of chordates prior to the gonadotropin-dependent HPG axis and are conserved not only in vertebrates but also in protovertebrates, ascidians. In addition, vertebrate ancestors might have acquired a new regulatory gonadotropin-dependent endocrine system, namely, the gonadotropin-dependent HPG axis, and employed this system for the regulation of subsequent follicle maturation and ovulation after the growth of secondary follicles to preantral follicles along with the development of the endocrine system and closed circulatory system.

In conclusion, we have substantiated an unprecedented secondary follicle growth process *via* the TK–JAK1–STAT3–COX-2–PGE2/PGF2α signaling cascade (Fig. 8). Our present data will contribute to the elucidation of the gonadotropin-independent follicle growth system in mammals and the evolution of the follicle growth system in chordates.

## Experimental procedures

### Animals

This study was approved by the Suntory Animal Ethics Committee and the Animal Care Committee of Nara Women’s University, and all animals were maintained in accordance with the committee guidelines for the care and use of laboratory animals. This study was approved by the Suntory Animal Ethics Committee (APRV000286, APRV000340, APRV000504, and APRV000667). *Tac1*-KO (B6. *Cg-Tac1<tm1Bbm/J; Tac1*^*−/−*^) mice were purchased from Jackson Laboratory, and C57BL/6 wildtype mice were purchased from SHIMIZU Laboratory Supplies. ICR (Institute for Cancer Research) mice were purchased from Japan SLC, Inc. All the mice were euthanized with CO_2_ asphyxiation.

### Quantification of follicle number

The ovaries were enucleated from four 3-week-old or 8-week-old C57BL/6 wildtype and *Tac1*-KO mice and fixed in Bouin’s fluid for 1 h. Subsequently, the ovaries were dehydrated with ethanol and embedded in paraffin. The paraffin-embedded ovaries were cut into 10-μm thick sections, followed by deparaffinization and rehydration. The sections were stained with hematoxylin and eosin, and images were obtained with an Axio Imager A2 microscope (Carl Zeiss). We precisely counted the follicles present in the ovarian section images. We counted preantral or more mature follicles (greater than 150 μm diameter) in the ovaries from each 3-week-old mouse or antral follicles (greater than 300 μm in diameter) in the ovaries from each 8-week-old mouse.

### Measurement of gonadotropin levels in mouse serum

Blood samples (100–200 μl) were collected from 3-week-old *Tac1*-KO mice and C57BL6 wildtype mice and incubated for 2 h at room temperature. Subsequently, the blood samples were centrifuged at 800*g* for 10 min to isolate and collect the serum. Serum gonadotropin levels were assessed by a Mouse LH ELISA Kit and Mouse FSH ELISA Kit (MyBioSource).

### Total RNA extraction and first-strand complementary DNA synthesis

Total RNA was extracted from the pituitary glands of 3-week-old *Tac1*-KO mice and C57BL6 wildtype mice using an RNeasy Plus Mini Kit (Qiagen) and reverse-transcribed to template complementary DNA at 50 °C for 50 min using an oligo dT anchor primer and SuperScript III Reverse Transcriptase (Life Technologies). Similarly, total RNA was extracted from cultured ovaries or collagen-embedded secondary follicles cultured in a three-dimensional culture system and reverse-transcribed as described previously. The obtained RNA was used for the custom service for gene expression service (Takara-Bio; accession no.: GSE213246) and the following experiments.

### Real-time PCR

Real-time PCR was performed using a CFX96 Real-time System and SsoAdvanced Universal SYBR Green Supermix (Bio-Rad Laboratories). The total volume of the real-time PCR mixture was 20 μl, consisting of 100 ng of template complementary DNA, 500 nM primer, and 10 μl of SYBR Green Master Mix solution. The real-time PCR amplification protocol was as follows: at 95 °C for 30 s, 44 cycles at 95 °C for 15 s and at 60 °C for 30 s. Melting curve analyses of the amplified PCR products were performed to confirm the absence of primer dimers. To evaluate the gene expression levels, we used the ΔΔCt method, which represents the difference in the expression of target genes in the pituitary gland between wildtype mice and *Tac1*-KO mice. The cycle threshold (Ct) value represents the PCR cycle number at which the PCR product was amplified at the determined level, and ΔCt represented the difference between Ct values determined from the PCR products prepared from the wildtype mouse and *Tac1*-KO mouse pituitary gland samples. Subsequently, ΔΔCt was calculated using the ΔCt of the *GAPDH* gene in the wildtype mouse pituitaries and *Tac1*-KO mouse pituitaries to normalize the target gene expression results. The primers used for real-time PCR were designed using the Primer-blast web tool (https://www.ncbi.nlm.nih.gov/tools/primer-blast/), and their sequences are shown in Table S3. Similarly, we analyzed the difference in the expression levels of target genes between untreated ovaries and chemically treated ovaries or secondary follicles using the ΔΔCt method. ΔCt shows the difference between Ct values calculated from the PCR products prepared from the untreated and chemically treated ovaries or secondary follicles. The ΔΔCt was calculated using the ΔCt of the *β-actin* gene in the untreated and chemically treated ovaries to normalize the target gene expression results. Relative expression score was calculated as 2^−ΔΔCT^. The primer sequences are shown in Table S3.

### Immunohistochemistry

The ovaries from 2-week-old mice were fixed at 25 °C for 15 min in Bouin fluid. The fixed ovaries were embedded in paraffin and cut into 7-μm sections. Immunostaining using various primary antibodies (Table S4) was performed as previously described (24). The immunoreactivity was visualized with an indirect immunofluorescence technique using secondary antibodies (Alexa 488 donkey anti-rabbit IgG and Alexa 568 donkey anti-goat IgG; Life Technologies) diluted with blocking buffer (1:500 dilution; v/v). Coverslips were mounted in FluorSave mounting medium (Merck), and the tissue slides were viewed using an Olympus BX51 photomicroscope (Olympus) equipped with epifluorescence. Because autofluorescence was detected in the ovarian sections, a WIB long-pass filter cube (Olympus) was used for observation.

### Three-dimensional follicle culture system

Mouse secondary follicles were cocultured with theca/interstitial cells using collagen gel (Cellmatrix Type I-A; Nitta Gelatine, Inc) as described in previous reports (37, 50). In brief, 0.2% collagen gel containing 10% fetal bovine serum (Thermo Fisher Scientific, Inc), 100 U/ml penicillin, 0.1 mg/ml streptomycin (Nacalai Tesque, Inc), and Dulbecco's modified Eagle's medium (Nissui Pharmaceutical Co, Ltd) was used to culture the secondary follicles. Approximately 20 to 30 secondary follicles that were 100 μm in diameter and 6 × 10^4^/well-theca/interstitial cells were cocultured with each ligand at 37 °C in 5% CO_2_ in air and 100% humidity for 5 days in a 96-well plate. Morphological changes in each follicle were observed using an inverted Olympus CK2 microscope (Olympus). The short and long diameters of the oocytes and follicles were measured using the image analysis tool ImageJ (https://imagej.nih.gov/ij/). Oocyte and follicle growth were calculated by determining the changes in diameter between oocytes and follicles cultured for 0 and 5 days.

### *In vitro* culture of ovaries

Mouse ovaries were cultured as previously reported (50). In brief, ovaries isolated from 2-week-old female mice were longitudinally cut into two symmetrical pieces to minimize experimental errors resulting from the use of different ovaries. The mouse ovaries were incubated on a Millicell Cell Culture Insert (12 mm, polycarbonate, 0.4 μm; Merck) with culture medium containing each chemical at 37 °C in 5% CO_2_ in air and 100% humidity. The chemicals used for *in vitro* ovarian organ culture are listed in Table S5.

### Statistical analysis

The results are shown as the mean ± SEM. Data were analyzed by Student’s *t* test with Welch’s correction or one-way ANOVA and Tukey’s multiple comparison test. Differences were considered significant at *p* < 0.05.

## Data availability

The gene expression profiles of mouse ovaries treated with TKR ligands have been deposited in the Gene Expression Omnibus database under accession no. GSE213246.

## Supporting information

This article contains supporting information.

## Conflict of interest

The authors declare that they have no conflicts of interest with the contents of this article.

## Supporting information

Supporting informaion-revised 2 clean
