# Supplementary material for: Ovarian tachykinin signaling system induces the growth of secondary follicles during the gonadotropin-independent process
Source: J Biol Chem. 2025 Mar 3;301(4):108375. doi: 10.1016/j.jbc.2025.108375 (PMC11999619; doi:10.1016/j.jbc.2025.108375)
Supplement: Supporting informaion-revised 2 clean [file mmc1.docx]

**Supporting Information for**

Ovarian tachykinin signaling system induces the growth of secondary follicles during the gonadotropin-independent process.

Tsuyoshi Kawada, Masato Aoyama, Shin Matsubara, Tomohiro Osugi, Akira Shiraishi, Tsubasa Sakai, Shinji Kirimoto, Satsuki Nakaoka, Yuki Sugiura, Keiko Yasuda, Honoo Satake*

*Correspondence to: Honoo Satake

Email: satake@sunbor.or.jp

**This PDF file includes:**

Supplementary Materials and Methods

Figures S1 to S5

Tables S1 to S6

Legends for Movies S1 to S4

**Other supporting materials for this manuscript include the following:**

Movies S1 to S4

Supporting Information Text

**SI Materials and Methods**

***Immunoelectron microscopy***

The ovaries from two-week-old mice were fixed at room temperature for 1 hr with 2% paraformaldehyde: 2% glutaraldehyde: 0.1 M phosphate buffer (pH 7.4). Anti-Substance P Polyclonal Antibody (bs-0065R-TR: Bioss Inc, Woburn, MA, USA) was used with the observation of immunoelectron microscopy. Immunoelectron microscopy observations were conducted by Tokai Electron Microscopic Analysis (Nagoya, Japan).

***DNA microarray analysis***

Total RNA was extracted from ovary halves cultured with 1 μM TACR1 agonist ([Sar9, Met(O2)11]-SP), TACR2 agonist (GR-64349), TACR3 agonist (succinyl-[Asp, N-Me-phe8]sp.6-11), TACR1 antagonist (L-703,606), TACR2 antagonist (GR-94800), or TACR3 antagonist (SB218795) (Supplementary Table 3) at 37 °C in 5% CO_2_ and 100% humidity for 3 days *in vitro* as stated above. We ordered a DNA microarray analysis using the RNA and a SurePrint G3 Mouse GE 8 x 60K Microarray chip from Takara Bio (Otsu, Japan). Fluorescent labelling and hybridization were performed using a Low Input Quick Amp Labelling Kit One-Colour (Agilent Technologies, Santa Clara, CA, USA) and a Gene Expression Hybridization Kit (Agilent Technologies). Array images were acquired using an Agilent laser scanner G2565CA (Agilent Technologies) and analysed with Agilent Feature Extraction software. The gene expression profiles have been deposited in the Gene Expression Omnibus database under accession no. GSE213246.

***Detection of prostaglandins using liquid chromatography‒mass spectrometry***

Fifty-two ovaries were collected from 2-week-old mice and longitudinally cut into symmetrical half-pieces. These ovary halves were cultured with 1 μM TACR1, TACR2, or TACR3 agonists at 37 °C in 5% CO_2_ in air and 100% humidity for 1 day. Prostaglandins were extracted and purified from the ovary pieces as previously reported (49). Briefly, cultured ovary pieces were frozen in liquid nitrogen and homogenized in methanol/formic acid solution (100:0.2) containing deuterium-labelled PGs as a control. The homogenate was diluted with water/formic acid solution (100:0.03) and applied to a solid-phase column (Oasis HLB cartridge; Waters, Milford, MA, USA). After washing with water/formic acid solution (100:0.03), water/ethanol/formic acid solution (90:10:0.03), and petroleum ether, the samples were eluted with methanol/formic acid solution (100:0.2). The resultant eluates were evaporated with a vacuum concentrator and dissolved in methanol (Kita et al 2005).

The dissolved samples were measured by liquid chromatography/mass spectrometry (LC‒MS) using a triple-quadrupole mass spectrometer equipped with an electrospray ionization (ESI) ion source (LCMS-8040, Shimadzu Corporation, Kyoto, Japan). The samples were resolved on a reversed-phase column (Kinetex C8, 2.1 × 150 mm, 2.6 μm; Phenomenex, Torrance, CA) using acetonitrile solution with 0.1% formic acid at a ﬂow rate of 0.4 ml/min and a column temperature of 40 °C. The deuterium-labelled standards were dissolved in the sample matrix (i.e., ovarian extract), and measured by LC‒MS as stated above.

**Ref.**

Kita, Y., Takahashi, T., Uozumi, N., Shimizu, T. (2005) A multiplex quantitation method for eicosanoids and platelet-activating factor using column-switching reversed-phase liquid chromatography-tandem mass spectrometry. *Anal Biochem*. 342,134-43.

***Total RNA extraction and first-strand cDNA synthesis***

Total RNA was extracted from 2-week-old mouse ovaries or secondary follicles using an RNeasy Plus Mini Kit (Qiagen; Hilden, Germany) and reverse-transcribed to template cDNA at 50 °C for 50 min using oligo dT anchor primers and SuperScript^TM^ III Reverse Transcriptase (Life Technology; Waltham, MA, USA).

***Reverse Transcription PCR (RT-PCR)***

PCR products of *prostaglandin (PG) synthase* genes and *PG receptor* genes were amplified using 0.5 μl of template cDNA solution, gene-specific primers (Supplementary Table 6), Taq^Ex^ polymerase (Takara; Shiga, Japan), and a thermal cycler (Model GeneAmp PCR System 9600; Applied Biosystems, Waltham, MA, USA). The PCR protocol was as follows: at 94 °C for 3 min, and 30 cycles (PG synthases) or 40 cycles (PG receptors) at 94 °C for 30 sec, at 60 °C for 30 sec, and at 72 °C for 30 sec. Likewise, PCR products of *JAK* genes and *STAT* genes were amplified with the following PCR protocol: 94 °C for 1 min, 30 cycles at 94 °C for 30 sec, at 58 °C for 30 sec, and at 72 °C for 1 min. Each PCR product was electrophoresed with 1.5% agarose gel.

***Real-time PCR***

A 100 ng aliquot of DNase-treated total RNA isolated from mouse ovariess was used for first-strand

cDNA synthesis. Real-time PCR was performed using a CFX96 Real-time System and SsoAdvanced Universal SYBR Green Supermix (Bio-Rad laboratories, Hercules, CA) according to the manufacture’s instruction. The primers used are listed in Table 3. The induction level of gene expression was calculated from the ΔΔCt values using the mouse *β-actin* gene. Relative expression score was calculated as 2^-ΔΔCT^.

***Measurement of gonadotropin levels in mouse serum***

Blood samples **(**100–200 μl) were collected from 3-week-old *Tac1*-KO mice and C57BL6 wild-type mice and incubated for 2 hr at room temperature. Subsequently, the blood samples were centrifuged at 800 x g for 10 min, and the serum was collected. Serum gonadotropin levels were assessed by a Mouse LH ELISA Kit and Mouse FSH ELISA Kit (MyBioSource; San Diego, CA, USA).


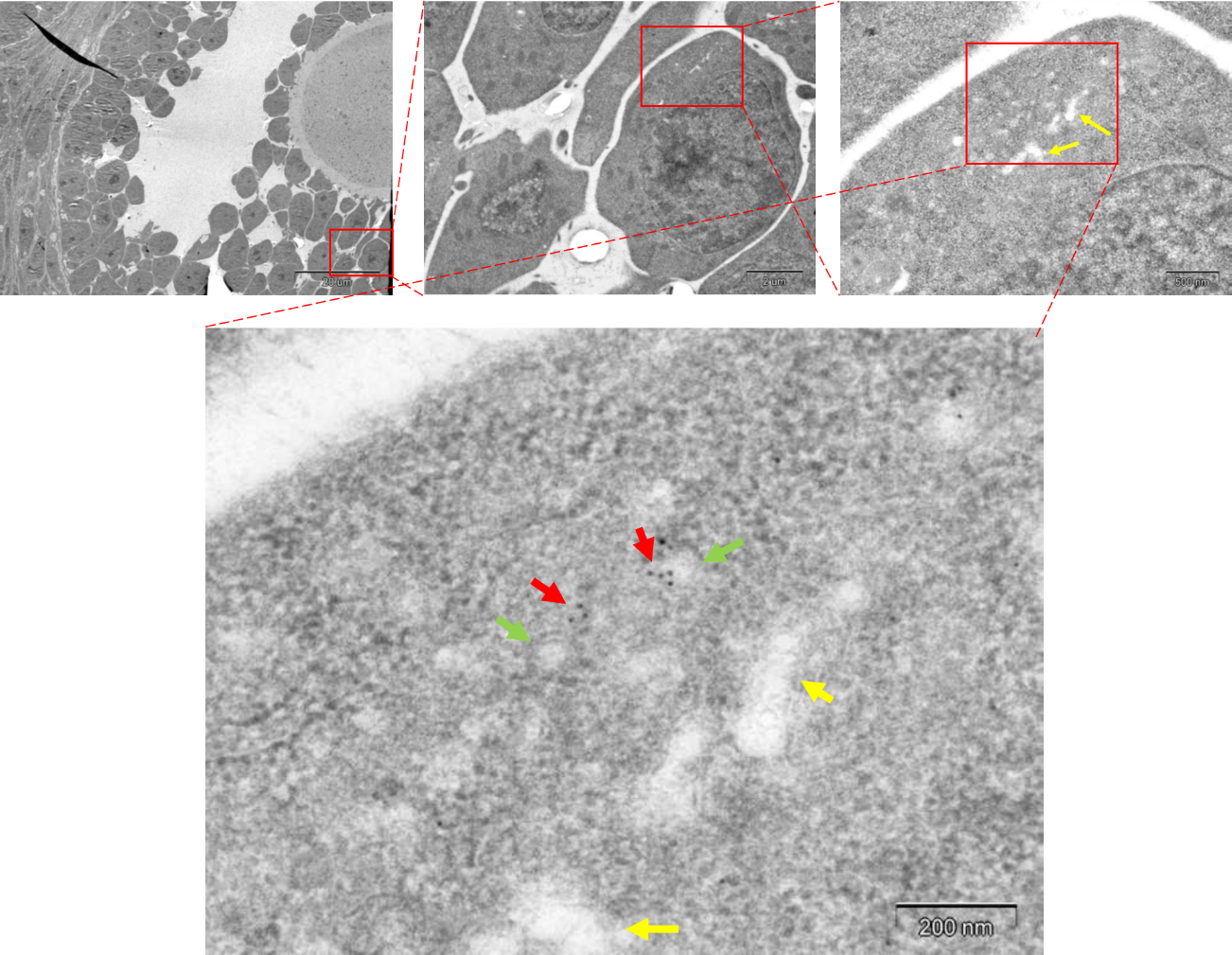


**Figure S1. Detection of substance P by immunoelectron microscopy using 2-week-old mouse ovary.** Immunological signals for SP in secretary granules are indicated by red arrows. Golgi apparatus and secretory vesicles are indicated by yellow and green arrows, respectively. Images within red boxes are progressively enlarged.


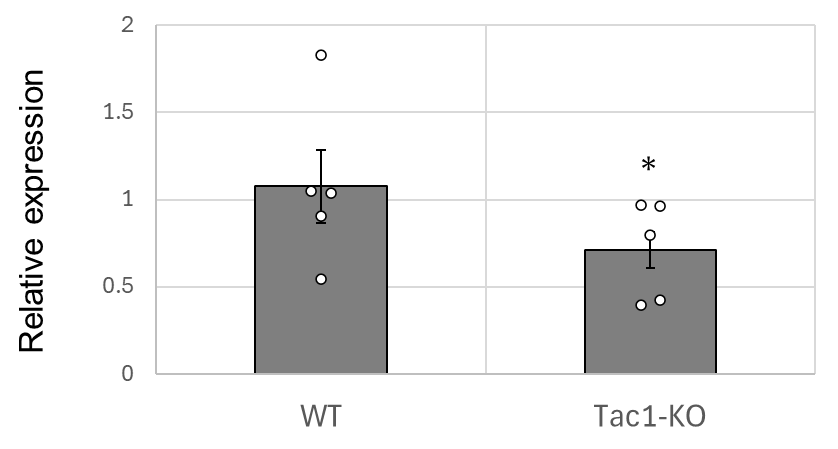


**Figure S2.** **Real-time PCR-based quantification of *COX-2* gene expression in ovaries of 2-week-old wild type and *Tac1-KO* mice.** The induction level of gene expression was calculated from the ΔΔCt values using the *β-actin* gene. Relative expression score was calculated as 2^-ΔΔCT^. Each point represents the mean ± SEM. A significant difference between WT and *Tac1-KO* mice is indicated by an asterisk (*P* < 0.05 according to the t test).

A


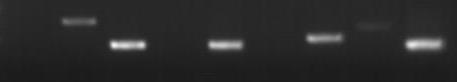


**1 2 3 4 5 6 7 8 9**

B


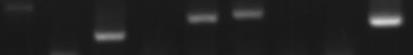


**1 2 3 4 5 6 7 8 9**

C


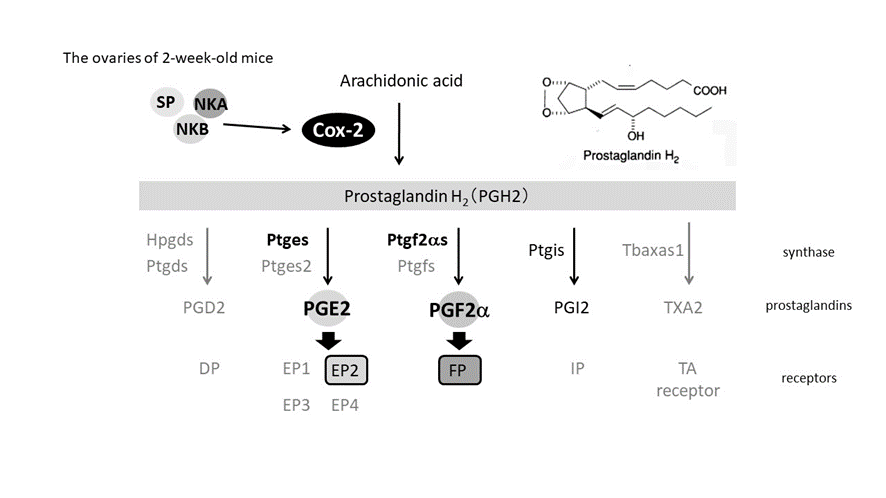


Figure S3. Gene expression of prostaglandin synthases and receptors in the ovaries of 2-week-old mice. (A) RT‒PCR products for prostaglandin synthase genes were generated from the RNA isolated from the mouse ovaries: (1) PGDS, haematopoietic prostaglandin D synthase (NM_019455); (2) PGD2S, prostaglandin D2 synthase (NM_008963); (3) PGES, prostaglandin E synthase (NM_022415); (4) PGES2, prostaglandin E synthase 2 (NM_133783); (5) PGF2αS, 13,14-dihydroprostaglandin F2 α synthase (NM_009658); (6) PGFS, prostamide/prostaglandin F synthase (NM_025582); (7) PGI2S, prostaglandin l2 synthase (NM_008968); (8) TBAS1, thromboxane A synthase 1 (NM_011539); and (9) β-actin (NM_007393). (B) RT‒PCR products for *prostaglandin receptor* genes are shown: (1) PGDR, prostaglandin D receptor (NM_008962); (2) PGER1, prostaglandin E receptor 1 (NM_013641); (3) PGER2 (EP2), prostaglandin E receptor 2 (NM_008964); (4) PGER3, prostaglandin E receptor 3 (NM_011196); PGER4 (EP4), (5) prostaglandin E receptor 4 (NM_008965); (6) PGER (FP), prostaglandin F receptor (NM_008966); (7) PGIR, prostaglandin I receptor (NM_008967); (8) TBA2R, thromboxane A2 receptor (NM_009325); and (9) b-actin (NM_007393). (C) Schematic image of prostaglandin synthesis and the interaction between prostaglandin and receptors in the ovaries of 2-week-old mice.

<insert page break here>
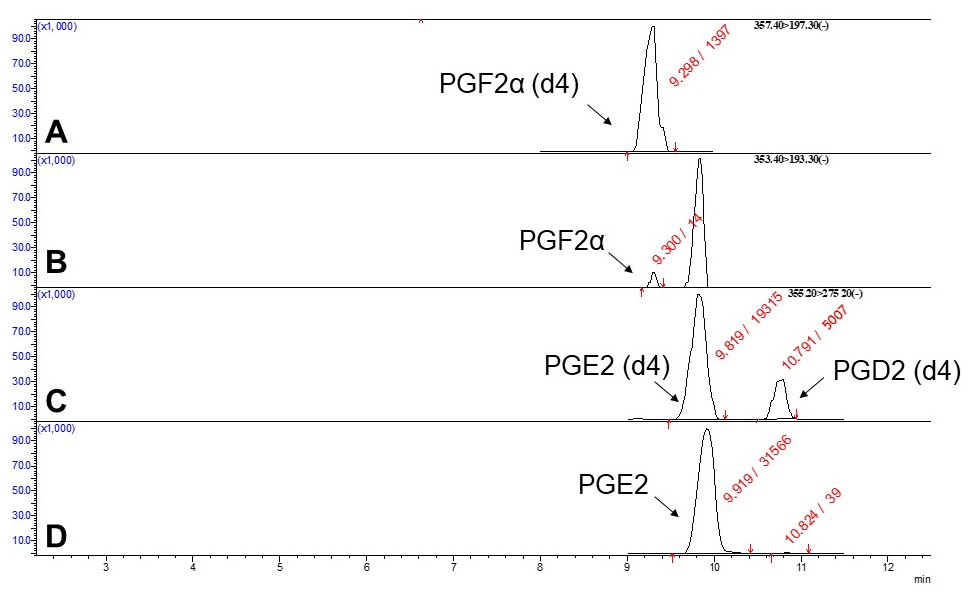
**Figure S4. Detection of prostaglandins using liquid chromatography‒mass spectrometry.** (A) Detection of deuterium-labelled PGF2α (PGF2α-d4) as a standard sample. (B) Detection of PGF2α in ovary extracts from 2-week-old mice. (C) Detection of deuterium-labelled PGE2 (PGE2(d4)) and PGD2 (PGD2(d4)) as standard samples. (D) Detection of PGE2 in ovary extracts from 2-week-old mice. The arrows indicate peaks originated from prostaglandins. Precursor ion peaks of PGE2 was detected as ion peaks of 351.2, while PGE2(d4) and PGD2(d4) were detected as ion peaks of 355.2. Likewise, precursor ion peaks of PGF2α and PGF2α(d4) were detected as ion peaks of 353.4 and 357.4. These numbers correspond to mass of each PG. Product ion peaks of PGE2 and PGE2(d4) were detected as ion peaks of 271.2 and 275.2, while precursor ion peaks of PGF2α and PGF2α(d4) were detected as ion peaks of 193.3 and 197.3. The values of each product ions peak are described in the upper right corner of each panel.


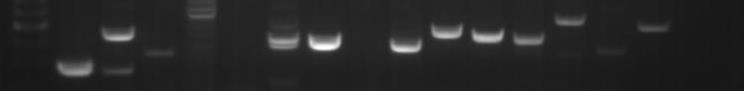


**1 2 3 4 5 6 7 8 9 10 11**

Figure S5. Gene expression of *JAK* and *STAT* genes in mouse secondary follicles. The following RT‒PCR products were generated from secondary follicle RNA for *JAK and STAT* gene expression analysis: (1) β-actin (NM_007393); (2) JAK1 (NM_146145); (3) JAK2 (NM_008413); (4) JAK3 (NM_010589); (5) STAT1 (NM_001205313); (6) STAT2 (NM_019963); (7) STAT3 (NM_213659); (8) STAT4 (NM_011487); (9) STAT5a (NM_011488); (10) STAT5b (NM_001113563); and (11) STAT6 (NM_009284).

Table S1. Quantification of gonadotropin levels s in the serum of 3-week-old mice via ELISA

Table S2. DNA microarray analysis of the *COX-2* (prostaglandin-endoperoxide synthase 2) gene in mouse ovaries treated with TK receptor (TKR) agonists or antagonists. Log2 ratio shows the ratio of gene expression levels in the ovaries of mice treated with TKR agonists to those in the ovaries of mice treated with TKR antagonists. NM_011198 is the GenBank accession number for the mouse *COX-2* gene.


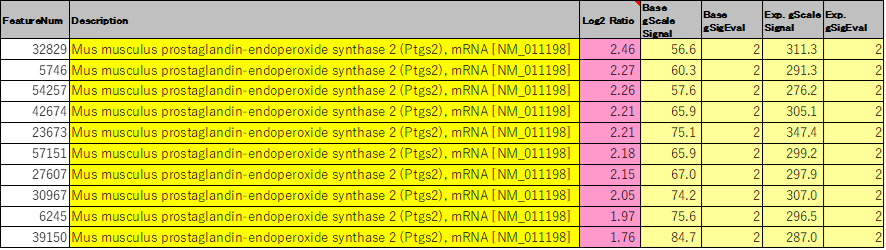


Table S3. List of primers for Real-time PCR

| **Table S4.** List of antibodies for immunohistochemistry | | | | | |
| --- | --- | --- | --- | --- | --- |
| Source | Antibody | Description (antigen) | Host | 1ry Dilution | 2ry Dilution |
| Peninsula Laboratories, LLC | Rabbit anti-SubstanceP | Mouse Substance P | rabbit | 1/50-1/100 | 1/500 |
| Peninsula Laboratories, LLC | Rabbit anti- Neurokinin A | Mouse Neurokinin A | rabbit | 1/50-1/100 | 1/500 |
| Santa Cruz | NeurokininB(H-51): sc-292436 | Mouse Neurokinin B | rabbit | 1/50-1/100 | 1/500 |
| Santa Cruz | NK-1R(L-15): sc-14115 | Mouse TACR1 | goat | 1/50-1/100 | 1/500 |
| Santa Cruz | NK-2R(M-48): sc-28951 | Mouse TACR2 | rabbit | 1/50-1/100 | 1/500 |
| Santa Cruz | NK-3R(H-105): sc-28952 | Mouse TACR3 | rabbit | 1/50-1/100 | 1/500 |
| Santa Cruz | Cox-2(M-19): sc-1747R | Mouse Cox-2 | rabbit | 1/50-1/100 | 1/500 |
| Santa Cruz | EP2(H-75): sc-20675 | Mouse EP2 | rabbit | 1/50-1/100 | 1/500 |
| Santa Cruz | PGF2aR(H-55): sc-67029 | Mouse FP | rabbit | 1/50-1/100 | 1/500 |

| **Table S5.** List of chemicals for three-dimensional follicle culture and *in vitro* ovarian organ culture | | | |
| --- | --- | --- | --- |
| Source | Chemicals (agonists, antagonists and inhibitors) | Description (target) | Final conc. |
| Sigma‒Aldrich | [Sar9,Met(O_2_)11]-SP | TACR1 agonist | 1 µM |
| Sigma‒Aldrich | GR-64349 | TACR2 agonist | 1 µM |
| Sigma‒Aldrich | Succinyl-[Asp, N-Me-phe8]sp.6-11 | TACR3 agonist | 1 µM |
| Sigma‒Aldrich | L-703,606 | TACR1 antagonist | 1 µM |
| Sigma‒Aldrich | GR-94800 | TACR2 antagonist | 1 µM |
| Sigma‒Aldrich | SB218795 | TACR3 antagonist | 1 µM |
| Sigma‒Aldrich | Celecoxib | COX-2 inhibitor | 1 µM |
| Cayman Chemical | NS-398 | COX-2 inhibitor | 5 µM |
| Santa Cruz | Oxaprozin | COX-1 inhibitor | 2.2 µM |
| Sigma‒Aldrich | Butaprost | EP2 agonist | 1 µM |
| Cayman Chemical | CAY10598 | EP4 agonist | 10 µM |
| Cayman Chemical | 17-phenyl trinor PGF2α | FP agonist | 1 µM |
| Cayman Chemical | AH6809 | EP2 antagonist | 50 µM |
| Cayman Chemical | GW627368X | EP4 antagonist | 1 µM |
| Cayman Chemical | AL8810 | FP antagonist | 10 µM |
| Cell Signaling Technology | PD98059 | ERK1/2 inhibitor | 100 µM |
| Cell Signaling Technology | SB202190 | p38 inhibitor | 100 µM |
| SANTA CRUZ | JAK Inhibitor I | JAK1, 2, 3 inhibitor | 40 µM |
| Adooq Bioscience | Pacritinib | JAK2, TYK2 inhibitor | 50 nM |
| Sigma‒Aldrich | Tofacitinib citrate | JAK3 inhibitor | 1 nM |
| Abcam | Fludarabine (FAMP) | STAT1 inhibitor | 5.4 nM |
| Abcam | Cryptotanshinone | STAT3 inhibitor | 50 µM |
| Abcam | IQDMA | STAT5 inhibitor | 8 µM |
| Cayman Chemical | AS1517499 | STAT6 inhibitor | 10 µM |

Table S6. List of primers for RT-PCR

Movie S1 (separate file). Movie of the ovaries of 3-week-old wild-type mice corresponding to the results shown in Fig. 1A and Table 1. Follicles more than 150 μm in diameter are indicated by numbers, and the diameter of the black circles is 150 μm.

Movie S2 (separate file). Movie of the ovaries of 3-week-old *Tac1*-KO mice corresponding to the results shown in Fig. 1A and Table 1. Follicles more than 150 μm in diameter are indicated by numbers, and the diameter of the black circles is 150 μm.

Movie S3 (separate file). Movie of the ovaries of 8-week-old wild-type mice corresponding to the results shown in Fig. 1B and Table 1. Follicles more than 300 μm in diameter are indicated by numbers, and the diameter of the black circles is 300 μm.

Movie S4 (separate file). Movie of the ovaries of 8-week-old *Tac1*-KO mice corresponding to the results shown in Fig. 1B and Table 1. Follicles more than 300 μm in diameter are indicated by numbers, and the diameter of the black circles is 300 μm.
